# Supplementary material for: Using the Power of Junctional Adhesion Molecules Combined with the Target of CAR-T to Inhibit Cancer Proliferation, Metastasis and Eradicate Tumors
Source: Biomedicines. 2022 Feb 4;10(2):381. doi: 10.3390/biomedicines10020381 (PMC8962422; doi:10.3390/biomedicines10020381)
Supplement: Supplementary file 1 [file biomedicines-10-00381-s001.zip › biomedicines-1555784-supplementary.pdf]

Supplementary File S1

DNA sequence of plasmid pET28a CM19XA, followed by amino acid sequence of CM19XA used in this study.

pET28a CM19XA

DNA sequence of the plasmid:

NcoI, ATG start

NdeI, starts CM19XA

Stop codon and XhoI, end of CM19XA

ACTATATCCGGATTGGCGAATGGGACGCGCCCTGTAGCGGCGCATTAAGCGCGGCGGGTGTGGTGGTTAC
GCGCAGCGTGACCGCTACACTTGCCAGCGCCCTAGCGCCCGCTCCTTTCGCTTTCTTCCCTTCCTTTCTC
GCCACGTTCGCCGGCTTTCCCCGTCAAGCTCTAAATCGGGGGCTCCCTTTAGGGTTCCGATTTAGTGCTT
TACGGCACCTCGACCCCAAAAAACTTGATTAGGGTGATGGTTCACGTAGTGGGCCATCGCCCTGATAGAC
GGTTTTTCGCCCTTTGACGTTGGAGTCCACGTTCTTTAATAGTGGACTCTTGTTCCAAACTGGAACAACA
CTCAACCCTATCTCGGTCTATTCTTTTGATTTATAAGGGATTTTGCCGATTTCGGCCTATTGGTTAAAAA
ATGAGCTGATTTAACAAAAATTTAACGCGAATTTTAACAAAATATTAACGCTTACAATTTAGGTGGCACT
TTTCGGGGAAATGTGCGCGGAACCCCTATTTGTTTATTTTTCTAAATACATTCAAATATGTATCCGCTCA
TGAATTAATTCTTAGAAAAACTCATCGAGCATCAAATGAAACTGCAATTTATTCATATCAGGATTATCAA
TACCATATTTTTGAAAAAGCCGTTTCTGTAATGAAGGAGAAAACTCACCGAGGCAGTTCCATAGGATGGC
AAGATCCTGGTATCGGTCTGCGATTCCGACTCGTCCAACATCAATACAACCTATTAATTTCCCCTCGTCA
AAAATAAGGTTATCAAGTGAGAAATCACCATGAGTGACGACTGAATCCGGTGAGAATGGCAAAAGTTTAT
GCATTTCTTTCCAGACTTGTTCAACAGGCCAGCCATTACGCTCGTCATCAAAATCACTCGCATCAACCAA
ACCGTTATTCATTCGTGATTGCGCCTGAGCGAGACGAAATACGCGATCGCTGTTAAAAGGACAATTACAA
ACAGGAATCGAATGCAACCGGCGCAGGAACACTGCCAGCGCATCAACAATATTTTCACCTGAATCAGGAT
ATTCTTCTAATACCTGGAATGCTGTTTTCCCGGGGATCGCAGTGGTGAGTAACCATGCATCATCAGGAGT
ACGGATAAAATGCTTGATGGTCGGAAGAGGCATAAATTCCGTCAGCCAGTTTAGTCTGACCATCTCATCT
GTAACATCATTGGCAACGCTACCTTTGCCATGTTTCAGAAACAACTCTGGCGCATCGGGCTTCCCATACA
ATCGATAGATTGTCGCACCTGATTGCCCGACATTATCGCGAGCCCATTTATACCCATATAAATCAGCATC
CATGTTGGAATTTAATCGCGGCCTAGAGCAAGACGTTTCCCGTTGAATATGGCTCATAACACCCCTTGTA
TTACTGTTTATGTAAGCAGACAGTTTTATTGTTCATGACCAAAATCCCTTAACGTGAGTTTTCGTTCCAC
TGAGCGTCAGACCCCGTAGAAAAGATCAAAGGATCTTCTTGAGATCCTTTTTTTCTGCGCGTAATCTGCT
GCTTGCAAACAAAAAAACCACCGCTACCAGCGGTGGTTTGTTTGCCGGATCAAGAGCTACCAACTCTTTT
TCCGAAGGTAACTGGCTTCAGCAGAGCGCAGATACCAAATACTGTCCTTCTAGTGTAGCCGTAGTTAGGC
CACCACTTCAAGAACTCTGTAGCACCGCCTACATACCTCGCTCTGCTAATCCTGTTACCAGTGGCTGCTG
CCAGTGGCGATAAGTCGTGTCTTACCGGGTTGGACTCAAGACGATAGTTACCGGATAAGGCGCAGCGGTC
GGGCTGAACGGGGGGTTCGTGCACACAGCCCAGCTTGGAGCGAACGACCTACACCGAACTGAGATACCTA
CAGCGTGAGCTATGAGAAAGCGCCACGCTTCCCGAAGGGAGAAAGGCGGACAGGTATCCGGTAAGCGGCA
GGGTCGGAACAGGAGAGCGCACGAGGGAGCTTCCAGGGGGAAACGCCTGGTATCTTTATAGTCCTGTCGG
GTTTCGCCACCTCTGACTTGAGCGTCGATTTTTGTGATGCTCGTCAGGGGGGCGGAGCCTATGGAAAAAC
GCCAGCAACGCGGCCTTTTTACGGTTCCTGGCCTTTTGCTGGCCTTTTGCTCACATGTTCTTTCCTGCGT
TATCCCCTGATTCTGTGGATAACCGTATTACCGCCTTTGAGTGAGCTGATACCGCTCGCCGCAGCCGAAC
GACCGAGCGCAGCGAGTCAGTGAGCGAGGAAGCGGAAGAGCGCCTGATGCGGTATTTTCTCCTTACGCAT
CTGTGCGGTATTTCACACCGCAATGGTGCACTCTCAGTACAATCTGCTCTGATGCCGCATAGTTAAGCCA

GTATACACTCCGCTATCGCTACGTGACTGGGTCATGGCTGCGCCCCGACACCCGCCAACACCCGCTGACG
CGCCCTGACGGGCTTGTCTGCTCCCGGCATCCGCTTACAGACAAGCTGTGACCGTCTCCGGGAGCTGCAT
GTGTCAGAGGTTTTCACCGTCATCACCGAAACGCGCGAGGCAGCTGCGGTAAAGCTCATCAGCGTGGTCG
TGAAGCGATTCACAGATGTCTGCCTGTTCATCCGCGTCCAGCTCGTTGAGTTTCTCCAGAAGCGTTAATG
TCTGGCTTCTGATAAAGCGGGCCATGTTAAGGGCGGTTTTTTCCTGTTTGGTCACTGATGCCTCCGTGTA
AGGGGGATTTCTGTTCATGGGGGTAATGATACCGATGAAACGAGAGAGGATGCTCACGATACGGGTTACT
GATGATGAACATGCCCGGTTACTGGAACGTTGTGAGGGTAAACAACTGGCGGTATGGATGCGGCGGGACC
AGAGAAAAATCACTCAGGGTCAATGCCAGCGCTTCGTTAATACAGATGTAGGTGTTCCACAGGGTAGCCA
GCAGCATCCTGCGATGCAGATCCGGAACATAATGGTGCAGGGCGCTGACTTCCGCGTTTCCAGACTTTAC
GAAACACGGAAACCGAAGACCATTCATGTTGTTGCTCAGGTCGCAGACGTTTTGCAGCAGCAGTCGCTTC
ACGTTCGCTCGCGTATCGGTGATTCATTCTGCTAACCAGTAAGGCAACCCCGCCAGCCTAGCCGGGTCCT
CAACGACAGGAGCACGATCATGCGCACCCGTGGGGCCGCCATGCCGGCGATAATGGCCTGCTTCTCGCCG
AAACGTTTGGTGGCGGGACCAGTGACGAAGGCTTGAGCGAGGGCGTGCAAGATTCCGAATACCGCAAGCG
ACAGGCCGATCATCGTCGCGCTCCAGCGAAAGCGGTCCTCGCCGAAAATGACCCAGAGCGCTGCCGGCAC
CTGTCCTACGAGTTGCATGATAAAGAAGACAGTCATAAGTGCGGCGACGATAGTCATGCCCCGCGCCCAC
CGGAAGGAGCTGACTGGGTTGAAGGCTCTCAAGGGCATCGGTCGAGATCCCGGTGCCTAATGAGTGAGCT
AACTTACATTAATTGCGTTGCGCTCACTGCCCGCTTTCCAGTCGGGAAACCTGTCGTGCCAGCTGCATTA
ATGAATCGGCCAACGCGCGGGGAGAGGCGGTTTGCGTATTGGGCGCCAGGGTGGTTTTTCTTTTCACCAG
TGAGACGGGCAACAGCTGATTGCCCTTCACCGCCTGGCCCTGAGAGAGTTGCAGCAAGCGGTCCACGCTG
GTTTGCCCCAGCAGGCGAAAATCCTGTTTGATGGTGGTTAACGGCGGGATATAACATGAGCTGTCTTCGG
TATCGTCGTATCCCACTACCGAGATATCCGCACCAACGCGCAGCCCGGACTCGGTAATGGCGCGCATTGC
GCCCAGCGCCATCTGATCGTTGGCAACCAGCATCGCAGTGGGAACGATGCCCTCATTCAGCATTTGCATG
GTTTGTTGAAAACCGGACATGGCACTCCAGTCGCCTTCCCGTTCCGCTATCGGCTGAATTTGATTGCGAG
TGAGATATTTATGCCAGCCAGCCAGACGCAGACGCGCCGAGACAGAACTTAATGGGCCCGCTAACAGCGC
GATTTGCTGGTGACCCAATGCGACCAGATGCTCCACGCCCAGTCGCGTACCGTCTTCATGGGAGAAAATA
ATACTGTTGATGGGTGTCTGGTCAGAGACATCAAGAAATAACGCCGGAACATTAGTGCAGGCAGCTTCCA
CAGCAATGGCATCCTGGTCATCCAGCGGATAGTTAATGATCAGCCCACTGACGCGTTGCGCGAGAAGATT
GTGCACCGCCGCTTTACAGGCTTCGACGCCGCTTCGTTCTACCATCGACACCACCACGCTGGCACCCAGT
TGATCGGCGCGAGATTTAATCGCCGCGACAATTTGCGACGGCGCGTGCAGGGCCAGACTGGAGGTGGCAA
CGCCAATCAGCAACGACTGTTTGCCCGCCAGTTGTTGTGCCACGCGGTTGGGAATGTAATTCAGCTCCGC
CATCGCCGCTTCCACTTTTTCCCGCGTTTTCGCAGAAACGTGGCTGGCCTGGTTCACCACGCGGGAAACG
GTCTGATAAGAGACACCGGCATACTCTGCGACATCGTATAACGTTACTGGTTTCACATTCACCACCCTGA
ATTGACTCTCTTCCGGGCGCTATCATGCCATACCGCGAAAGGTTTTGCGCCATTCGATGGTGTCCGGGAT
CTCGACGCTCTCCCTTATGCGACTCCTGCATTAGGAAGCAGCCCAGTAGTAGGTTGAGGCCGTTGAGCAC
CGCCGCCGCAAGGAATGGTGCATGCAAGGAGATGGCGCCCAACAGTCCCCCGGCCACGGGGCCTGCCACC
ATACCCACGCCGAAACAAGCGCTCATGAGCCCGAAGTGGCGAGCCCGATCTTCCCCATCGGTGATGTCGG
CGATATAGGCGCCAGCAACCGCACCTGTGGCGCCGGTGATGCCGGCCACGATGCGTCCGGCGTAGAGGAT
CGAGATCTCGATCCCGCGAAATTAATACGACTCACTATAGGGGAATTGTGAGCGGATAACAATTCCCCTC
TAGAAATAATTTTGTTTAACTTTAAGAAGGAGATATACC**ATG**GGCAGCAGCCATCATCATCATCATCACA
GCAGCGGCCTGGTGCCGCGCGGCAGC**CATATG**GAAAACCTGTATTTCCAGGGGCCCGAGGAACCCCTGGT
GGTGAAAGTCGAGGAGGGCGATAACGCTGTCCTGCAATGCCTTAAGGGAACGTCTGACGGCCCCACACAA
CAATTAACATGGTCTCGCGAATCCCCCTTGAAGCCCTTTTTGAAACTGTCATTAGGCTTGCCCGGTCTTG
GGATCCACATGCGTCCATTAGCGATTTGGCTTTTCATCTTCAATGTCTCGCAACAAATGGGCGGTTTCTA
CCTTTGTCAACCTGGACCCCCTAGTGAAAAAGCGTGGCAACCCGGGTGGACTGTCAATGTCGAAGGATCT
GGCGAGCTTTTCCGTTGGAACGTAAGTGACTTAGGTGGACTGGGCTGCGGTCTTAAAAATCGCTCCAGCG
AGGGCCCAAGTAGTCCATCTGGCAAACTGATGTCGCCAAAGCTTTATGTTTGGGCAAAAGATCGCCCAGA
AATTTGGGAAGGTGAGCCGCCCTGCTTACCACCCCGTGATTCTTTGAACCAATCACTTAGCCAGGACTTA

ACTATGGCACCAGGGAGTACGCTTTGGTTGAGTTGCGGCGTTCCTCCTGATTCAGTCTCCCGCGGCCCTC
TGTCTTGGACGCACGTCCATCCAAAGGGCCCGAAGTCCCTGTTAAGCCTTGAGTTGAAAGATGACCGCCC
TGCTCGCGATATGTGGGTCATGGAAACGGGGTTGCTTTTACCGCGTGCCACAGCGCAAGATGCGGGAAAA
TATTACTGCCACCGCGGAAATTTAACGATGTCCTTCCATTTGGAGATCACCGCCCGTCCGGTATTGTGGC
ACTGGCTGCTGCGCACAGGGGGCTGGAAAGGGGGATCCGGTGGGTCTGGAGGAAGTGGCGGAAGTGGTGG
ATCTGGCGGTAGTTCCGTGACGGTCCACTCATCTGAGCCGGAAGTTCGCATTCCTGAAAACAACCCCGTA
AAACTGAGTTGCGCCTATTCAGGGTTTTCATCACCTCGCGTCGAATGGAAATTCGATCAAGGTGACACCA
CTCGCTTAGTGTGTTATAACAACAAAATCACGGCGTCATACGAGGATCGCGTTACTTTCTTACCAACAGG
CATCACCTTTAAATCTGTAACACGCGAGGATACAGGAACCTATACTTGTATGGTTTCGGAGGAGGGTGGG
AATTCGTACGGCGAGGTAAAAGTCAAATTGATTGTGTTAGTCCCGCCTTCAAAGCCCACGGTTAATATTC
CCTCGTCTGCTACAATTGGTAACCGCGCCGTTCTTACATGCTCCGAACAAGATGGTAGTCCGCCCTCCGA
ATATACATGGTTCAAGGACGGCATCGTAATGCCAACAAATCCGAAAAGTACACGCGCTTTCAGCAATTCG
TCATACGTCTTAAATCCCACCACCGGCGAATTGGTCTTCGATCCACTTTCAGCCTCTGACACTGGTGAGT
ACTCATGCGAGGCTCGTAATGGCTATGGTACGCCAATGACAAGTAACGCTGTACGCATGGAGGCGGTCGA
ACGCAACGTTGGAGTTGCCTGTGAGCAGAATCCCATCTACTGGGCGCGCTATGCGGACTGGTTATTCACG
ACACCCTTACTGCTGTTGGACTTAGCCCTTCTTGTTGACGCCGATGAGGGAACTGGGTGATGA**CTCGAG**C
ACCACCACCACCACCACTGAGATCCGGCTGCTAACAAAGCCCGAAAGGAAGCTGAGTTGGCTGCTGCCAC
CGCTGAGCAATAACTAGCATAACCCCTTGGGGCCTCTAAACGGGTCTTGAGGGGTTTTTTGCTGAAAGGA
GGA

Translation product of pEt28a CM19XA

```
        10         20         30         40         50         60
```

MGSSHHHHHH SSGLVPRGSH MENLYFQGPE EPLVVKVEEG DNAVLQCLKG TSDGPTQQLT

```
        70         80         90        100        110        120
WSRESPLKPF LKLSLGLPGL GIHMRPLAIW LFIFNVSQQM GGFYLCQPGP PSEKAWQPGW

       130        140        150        160        170        180
TVNVEGSGEL FRWNVSDLGG LGCGLKNRSS EGPSSPSGKL MSPKLYVWAK DRPEIWEGEP

       190        200        210        220        230        240
PCLPPRDSLN QSLSQDLTMA PGSTLWLSCG VPPDSVSRGP LSWTHVHPKG PKSLLSLELK

       250        260        270        280        290        300
DDRPARDMWV METGLLLPRA TAQDAGKYYC HRGNLTMSFH LEITARPVLW HWLLRTGGWK

       310        320        330        340        350        360
GGSGGSGGSG GSGGSGGSSV TVHSSEPEVR IPENNPVKLS CAYSGFSSPR VEWKFDQGDT

       370        380        390        400        410        420
TRLVCYNNKI TASYEDRVTF LPTGITFKSV TREDTGTYTC MVSEEGGNSY GEVKVKLIVL

       430        440        450        460        470        480
VPPSKPTVNI PSSATIGNRA VLTCSEQDGS PPSEYTWFKD GIVMPTNPKS TRAFSNSSYV

       490        500        510        520        530        540
LNPTTGELVF DPLSASDTGE YSCEARNGYG TPMTSNAVRM EAVERNVGVA CEQNPIYWAR

       550        560
YADWLFTTPL LLLDLALLVD ADEGTG
```

Number of amino acids: 566

Molecular weight: 61550.42
